# Association study in naturally infected helminth layers shows evidence for influence of interferon-gamma gene variants on *Ascaridia galli *worm burden

**DOI:** 10.1186/1297-9716-42-84

**Published:** 2011-07-12

**Authors:** Gesine Lühken, Matthias Gauly, Falko Kaufmann, Georg Erhardt

**Affiliations:** 1 Department of Animal Breeding and Genetics, Justus-Liebig University of Giessen, Ludwigstrasse 21B, 35390 Giessen, Germany; 2 Department of Animal Science, Georg-August University of Goettingen, Albrecht-Thaer-Weg 3, 37075 Goettingen, Germany

## Abstract

Single nucleotide polymorphisms (SNPs) in the genes for interleukin-4, -13 and interferon-gamma, and 21 additional SNPs which previously had been significantly associated with immune traits in the chicken, were genotyped in white and brown layer hens and analyzed for their association with helminth burden following natural infections. A nucleotide substitution located upstream of the promoter of the interferon-gamma gene was significantly associated with the log transformed number of *Ascaridia galli *in the brown layer line (genotype *CC*: 6.4 ± 1.0 worms; genotype *CT*: 11.7 ± 2.2 worms). Therefore, *IFNG *seems to be a promising candidate gene for further studies on helminth resistance in the chicken.

## Introduction, Methods, and Results

In the European Community, animal welfare issues and changes in consumer demands have resulted in a ban of conventional cages for laying hens from 2012 on (Council Directive 1999/74). This has resulted in an increased importance of floor husbandry systems and consequently in a renewed relevance of helminthoses [1]. The development of drug resistance in nematodes [2,3] and restrictions for the use of anthelmintics in food producing animals are two important aspects urging scientists to find alternative strategies for the control of gastrointestinal infections in laying hens. Estimated heritabilities and breed or line differences for immunological characteristics were not only shown in mammals but also in poultry [4,5]. Moreover, heritabilities estimated for parameters of susceptibility to helminthic infections, as mean worm or larvae counts [6-10], suggest that it is possible to select for helminth resistance in poultry.

Although immunity in birds is not as well understood as in mammals, it has been shown that as in mammals [11], helminth infection in chickens results in polarization towards a type 2 immune reaction, including augmented expression of interleukin-4 and interleukin-13 and diminished interferon-gamma expression [12]. In a single nucleotide polymorphism (SNP) study concerning innate and adaptive immune response across white and brown layer lines, 59 significant associations between immune traits and SNPs in immunological relevant genes were detected [13]; however, variants of interleukin-4 (*IL4*), interleukin-13 (*IL13*) and interferon-gamma (*IFNG*) genes were not included.

The aim of the present study was to determine genotypes of SNPs in the *IL4*, *IL13 *and *IFNG *genes and of 21 additional SNPs significantly associated with immune traits in white and brown commercial layer lines and to analyze their association with worm numbers resulting from a natural helminth infection in order to identify gene regions as promising candidates for further studies on parasite resistance in chickens.

Whole blood samples, numbers of adult worms of *Ascaridia galli*, *Heterakis gallinarum*, *Capillaria *spp. and tapeworms of 197 Lohmann Brown (LB) and 246 Lohmann Selected Leghorn (LSL) hens and pedigree data (sires) were available from a recent study conducted by Kaufmann et al. [6]. Briefly, in their experiment LB and LSL hens were reared under helminth-free conditions and kept afterwards together in a free range system. At the end of the laying period, hens were slaughtered and worms were counted according to the World Association for the Advancement of Veterinary Parasitology (WAAVP) guidelines. Whereas LB hens showed a significantly (*P *< 0.05) higher mean number of adult *H. gallinarum*, *Capillaria *spp. and tapeworms compared to LSL animals, the latter had a tendency towards a higher number of adult *A. galli *worms. The estimated heritabilities for worm burdens of the different helminths and of the total worm burden ranged from 0.11 to 0.69 in LB and from 0.01 to 0.30 in LSL. Further details are given by Kaufmann et al. [6]. DNA was extracted from whole blood samples of these 443 hens using the Invisorb Blood Mini HTS 96 Kit (Invitek, Berlin, Germany). Quality and quantity of DNA were checked after extraction using a NanoDrop spectrophotometer (NanoDrop Technologies, Wilmington, USA).

In a previous work, we sequenced the 5'-flanking and all coding regions of *IFNG*, *IL4 *and *IL13 *in 20 chickens, 10 each from the white and brown layer White Leghorn and New Hampshire breeds (unpublished). Among the identified SNPs, only those which were polymorphic in at least one of the breeds were selected for genotyping. Preferably, they were located in or near functional gene regions. Three of the selected SNPs had not been listed in the database of genetic variation [14] and therefore sequence information for those was sent to GenBank [GenBank:HQ888866-HQ888868]. Genotyping of two *IFNG *and three *IL4 *SNPs was done by PCR restriction-fragment-length-polymorphism (RFLP) analysis. For this purpose forward and reverse primers for *IFNG *(SNP in 5'-flanking region: 5'-tgaccccttaaccacatgatt-3' and 5'- tcttaaagcatggtcctggaa-3', 194 bp; SNP in exon 4: 5'- gcagttaagcctgagggatg-3' and 5'- cctcattcggtattttcaggtc-3', 462 bp) and for *IL4 *(SNPs in exon 1 and intron 1: 5'-acctcacggggagagaaagt-3' and 5'-tcgagctggctttcctctta-3', 554 bp; SNP in intron 3: 5'-tgctgttctaatccactcaagaa-3' and 5'-aaagctgctcccatcttttc-3', 725 bp) were used to amplify DNA fragments that were digested with appropriate restriction enzymes (Table 1) according to the manufacturers' (MBI Fermentas, St. Leon-Rot, Germany; New England Biolabs, Frankfurt, Germany) recommendations. The last nucleotide of the forward primer for the *IFNG *5'-flanking region was a mismatch in order to enable RFLP analysis by an amplification created restriction site [15].

**Table 1:** Numbers, locations, genotyping methods and *P*-values for allele frequency differences between lines of analyzed SNPs.

**SNP no**.	**rs or GenBank accession no**.	GGA^1^	gene symbols (gene region)	genotyping method^2^	*P *(allele frequencies between lines)
1	HQ888866	1	*IFNG *(5'-flanking region)	PCR-RFLP (*Hinf*I)	< 0.001

2	HQ888867	1	*IFNG *(exon 4, synonymous)	PCR-RFLP (*Mbo*II)	< 0.001

3	rs13526054	3	*IL17F *(exon, synonymous)	MALDI-TOF MS	< 0.001

4	rs14082130	3	*MAL *(intron)	MALDI-TOF MS	< 0.001

5	rs15458146	3	*IL17F *(exon, nonsynonymous)	MALDI-TOF MS	< 0.001

6	rs13520872	4	*SHROOM3 *(intron)	MALDI-TOF MS	< 0.001

7	rs13520980	4	*NUP54 *(intron)	MALDI-TOF MS	0.834

8	rs13521841	4	no gene	MALDI-TOF MS	< 0.001

9	rs15475503	4	*HTR2C *(intron)	MALDI-TOF MS	< 0.001

10	rs13586560	5	*ENTPD5 *(intron)	MALDI-TOF MS	< 0.001

11	rs13586776	5	*FLVCR2 *(intron)	MALDI-TOF MS	< 0.001

12	rs13755931	5	*SPTBN5 *(intron)	MALDI-TOF MS	0.007

13	rs15669480	5	*TOLLIP *(exon, synonymous)	MALDI-TOF MS	< 0.001

14	rs14580491	6	*CXCL12 *(intron)	MALDI-TOF MS	< 0.001

15	rs13596817	7	no gene	MALDI-TOF MS	< 0.001

16	rs13596877	7	no gene	MALDI-TOF MS	< 0.001

17	rs13599559	7	*SPOPL *(intron)	MALDI-TOF MS	< 0.001

18	HQ888868	13	*IL4 *(exon 1, synonymous)	PCR-RFLP (*Taa*I)	< 0.001

19	rs13505561	13	*IL4 *(intron 1)	PCR-RFLP (*Taa*I)	0.012

20	rs15709667	13	*IL4 *(intron 3)	PCR-RFLP (*Bcc*I)	< 0.001

21	rs14064765	13	*GMCSF *(5'-flanking region)	MALDI-TOF MS	0.248

22	rs14064896	13	*IRF1 *(3'-flanking region)	MALDI-TOF MS	< 0.001

23	rs15677371	13	no gene	MALDI-TOF MS	< 0.001

24	rs15677377	13	no gene	MALDI-TOF MS	< 0.001

25	rs15709642	13	*IL13 *(intron)	MALDI-TOF MS	< 0.001

26	rs15788216	16	*MHC, BLB1 *(exon, nonsynonymous)	MALDI-TOF MS	1.000

27	rs14119843	19	*HSPB1 *(3'-flanking region)	MALDI-TOF MS	< 0.001

^1^number of chicken chromosome (*Gallus gallus*); ^2^for PCR-RFLP, used restriction enzymes are given in parentheses; *CXCL12*, chemokine (C-X-C motif) ligand 12; *ENTPD5*, ectonucleoside triphosphate diphosphohydrolase 5; *FLVCR2*, feline leukemia virus subgroup C cellular receptor family, member 2; *GMCSF*, granulocyte-macrophage colony-stimulating factor; HSPB1, heat shock 27kDa protein 1; *HTR2C*, 5-hydroxytryptamine (serotonin) receptor 2C; *IFNG*, interferon gamma; *IL4*, interleukin 4; *IL13*, interleukin 13; *IL17F*; interleukin 17F; *IRF1*, interferon regulatory factor 1; *MAL*, mal, T-cell differentiation protein; *MHC *(*BLB1*), major histocompatibility complex class II antigen B-F minor heavy chain; *NUP54*, nucleoporin 54kDa; *SHROOM3*, shroom family member 3; *SPOPL*, speckle-type POZ protein-like; *SPTBN5*, spectrin, beta, non-erythrocytic 5; *TOLLIP*, toll interacting protein.

SNP genotypes were discriminated after electrophoresis of the digested PCR products on agarose gels and ethidium bromide staining.

All other SNPs (*n *= 22) were genotyped with matrix-assisted laser desorption/ionization time of flight mass spectrometry (MALDI-TOF MS) by Eurofins Medigenomix GmbH, Martinsried, Germany, using the Sequenom Massarray iPLEX Gold System (Sequenom, San Diego, USA). Twenty-one SNPs were chosen from the study of Biscarini et al. [13]. All of these SNPs were significantly associated with at least one of the analyzed immune traits (production of natural antibodies against exo- and endo-antigens and of acquired antibodies, activation of classical and alternative complement pathways) with a *P*-value < 0.01 [13]. Furthermore, a nucleotide substitution in intron 1 of *IL13*, already recorded in the db SNP database (rs15709642), was included in the MALDI-TOF MS multiplex assay.

For all genotyped SNPs, the rs number or a GenBank accession number, the chromosomal and gene location and the genotyping method used are given in Table 1.

SNP allele frequencies were calculated from the genotypes obtained. The significance of differences between allele frequencies of the genotyped SNPs in the two lines were analyzed with a chi square test, or with a Fisher exact test if the smallest cell contained less than six cases. Worm numbers were log transformed [log(worm number + 10)] to get approximately normally distributed data, as done before by Kaufmann et al. [6]. Association studies were performed for each SNP - showing a minor allele frequency ≥ 5% per line - with each of the observed parasitological traits, using the following statistical model: y*_ij _*= μ + SNP*_i _*+ e*_ij_*, where *y_ij _*represents the observation for the animal j, with SNP genotype i; μ is the overall mean of the trait; SNP*_i _*is the effect of the SNP genotype, either AA, AB or BB; and *e_ij _*is the random residual effect. Association analysis was done within each line for all SNPs and additionally across lines for SNPs whose allele frequencies were not significantly (*P *< 0.05) different between the two lines.

**Figure 1:**
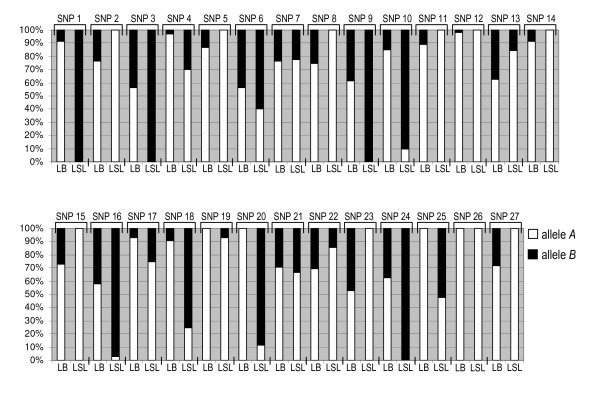
**Allele frequencies (%) of SNPs in Lohmann Brown (LB) and Lohmann Selected Leghorn (LSL) hens**. For rs or GenBank accession numbers of SNPs and their location on chromosomes and in genes see Table 1.

Allele frequencies of the 27 genotyped SNPs are shown in Figure 1 for each line. A total of 14 SNPs was monomorphic and 1 additional SNP had a minor allele frequency < 0.05 in LSL, whereas 3 SNPs were fixed and 3 showed a minor allele frequency < 0.05 in LB. SNP 26, located in the *BLB1 *region of the major histocompatibility complex (*MHC*), was fixed in both lines. SNP 12 showed a minor allele frequency < 0.05 in LB and was monomorphic in LSL. Therefore SNPs 12 and 26 were not included in association analyses in any of the two lines.

**Figure 2:**
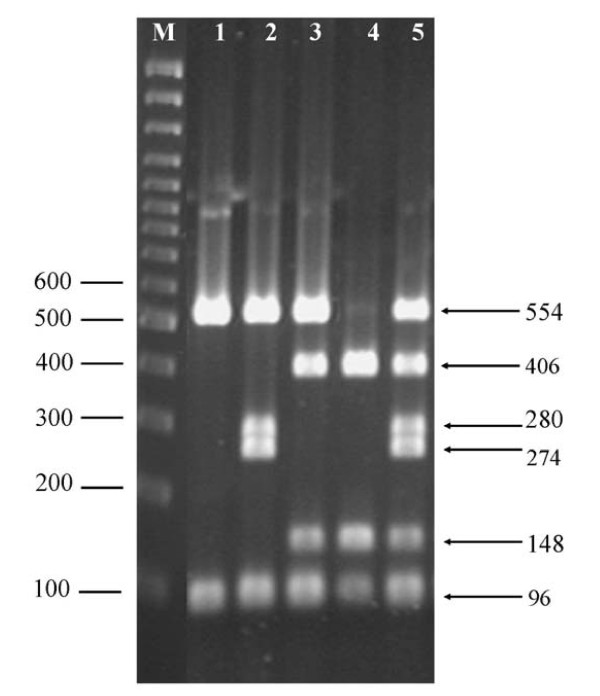
**Genotyping of chicken *IL4 *haplotypes (SNP exon 1 - SNP intron 1) by PCR-RFLP analysis**. PCR products from hens with different genotypes (1-5) digested with ***Taa ***I, separated by agarose gel electrophoresis and stained with ethidium bromide. 1 = ***G***-***G***/***G***-***G***, 2 = ***G***-***G***/***A***-***G***, 3 = ***G-G***/***G***-***C***, 4 = ***G***-***C***/***G***-***C***, 5 = ***G-C/A-G ***(554 bp fragment usually not completely digested in genotype 5)**. **M: 100 bp DNA size marker. Left numbers: marker sizes in bp. Right numbers: DNA fragment sizes in bp.

The SNPs in exon 1 and intron 1 of *IL4 *were genotyped by amplifying a single PCR product and digested with a single enzyme, enabling the demonstration of a total of 3 haplotypes (Figure 2). Only two of these haplotypes were identified in LB but all three in LSL (haplotype frequencies not shown).

Besides the fixed SNP 26, the allele frequencies of only two SNPs (7 and 21) were not significantly different between LB and LSL (Table 1). Therefore only those two SNPs were also analyzed for association with worm numbers across lines and not only within lines.

*P*-values resulting from analysis of the association of SNP genotypes with worm numbers of *A. galli*, *H. gallinarum*, *Capillaria *spp., tapeworms and total worm burden are given in Table 2. Three SNPs (1 and 21, located in the 5'-flanking region of *IFNG *and *GMCSF*, respectively, and SNP 4, an intronic nucleotide substitution in *MAL*) were significantly associated with one of the traits analyzed; whereas 7 SNPs showed a tendency towards significance for association with one or more of the traits (Table 2). Although only SNPs with a minor allele frequency < 5% were tested for association with the different parasitological traits, for some of the SNPs studied, the statistical significance or tendency towards significance of an association was obviously connected with a genotype only present in a small portion of hens (< 10%). This was also the case for the significant associations of SNPs 4 and 21 with parasitological traits.

**Table 2:** *P*-values for association of SNPs with worm numbers in LB and LSL or both (all).

**SNP no**.	line	*A. galli*	*H. gallinarum*	***Capillaria *ssp**.	tapeworms	all helminths
1	LB	**0.017**	0.175	0.356	0.289	0.287

2	LB	0.994	0.487	0.695	0.163	0.754

3	LB	0.375	0.977	0.129	0.408	0.842

4	LB	0.479	**0.052**	**0.077**	0.215	0.133

	LSL	0.186	0.472	0.540	0.992	0.280

5	LB	0.301	0.983	0.152	0.494	0.855

6	LB	0.304	0.439	0.105	0.980	0.680

	LSL	**0.068**	**0.082**	0.346	0.828	**0.063**

7	LB	0.838	0.892	0.913	0.757	0.916

	LSL	0.793	0.231	0.604	0.537	0.265

	all	0.984	0.673	0.659	0.886	0.716

8	LB	0.493	0.203	0.722	0.479	0.153

9	LB	**0.086**	0.515	0.154	0.693	0.316

10	LB	0.488	0.951	0.750	0.137	0.841

	LSL	0.489	0.672	0.872	0.667	0.823

11	LB	0.741	0.647	0.620	0.686	0.577

13	LB	0.687	0.215	0.926	0.743	0.165

	LSL	0.205	0.398	**0.067**	0.600	0.212

14	LB	0.535	0.306	0.365	0.734	0.487

15	LB	0.677	0.225	0.669	0.639	0.219

16	LB	0.359	0.609	0.827	0.604	0.796

	LSL	0.187	0.692	0.547	0.150	0.938

17	LB	0.500	0.126	0.141	0.216	0.319

	LSL	**0.081**	0.375	0.146	0.537	0.179

18	LSL	0.462	0.193	0.758	0.830	0.303

19	LSL	0.613	0.317	0.676	0.620	0.427

18-19	LSL	0.607	0.431	0.841	0.756	0.615

20	LSL	0.815	0.521	0.828	0.847	0.517

21	LB	0.960	0.322	0.875	**0.056**	0.529

	LSL	**0.090**	0.678	**0.061**	0.540	0.296

	all	0.418	0.435	0.856	**0.003**	0.500

22	LB	0.628	0.292	0.371	0.878	0.459

	LSL	0.605	0.182	0.337	0.789	0.200

23	LB	0.067	0.213	0.138	0.411	0.122

24	LB	0.930	0.938	0.327	0.207	0.623

25	LB	0.495	0.886	0.260	0.625	0.632

	LSL	0.337	**0.074**	**0.067**	0.413	0.113

27	LB	0.133	0.305	0.124	0.924	0.471

Significant associations (*P *< 0.05) and associations with a tendency towards significance (*P *< 0.1) are bold typed.

For SNP 1, showing significant association with the log transformed worm number of *A. galli *in LB, genotype *CC *was very frequent (83%), whereas genotype *CT *occurred in a lower frequency (17%). The average *A. galli *worm number was 6.4 ± 1.0 in LB hens with the genotype *CC*, whereas it was 11.7 ± 2.2 in hens with the genotype *CT*. As 10 of the 19 LB sires had only progeny with the *CC *genotype, the association analysis for SNP 1 regarding the number of *A. galli *in LB was repeated only with hens (*n *= 90) from the 9 other sires, resulting in a *P*-value of 0.011.

## Discussion

The higher number of monomorphic SNPs in the white layer line (52% of the SNPs analyzed) compared to the brown (11% of the SNPs analyzed) was in accordance with other studies [13,16] and can be attributed to the smaller number of incorporated breeds in white lines [16,17]. Nevertheless, we did not expect such a large difference since Biscarini et al. [13] reported only 6% more fixed loci in 5 white layer lines compared to 4 in the brown lines. The larger difference observed in the present study could be due to the smaller number of analyzed SNPs or a higher homozygosity of LSL and/or lower homozygosity of LB compared to the average of the white and brown layer lines analyzed by Biscarini [13]. Interestingly, the lower homozygosity in LB is - excluding the *A. galli *worm number- accompanied by higher heritabilities for worm numbers and at the same time significantly higher worm numbers, compared to LSL [6]. Among the SNPs which showed significant associations with parasitological traits, SNP 1 is the only one where this was not obviously linked to a very rare genotype. Genotypes *CC *and *CT *of SNP 1, a nucleotide substitution we previously identified in the *IFNG *5'-flanking region of New Hampshire and White Leghorn, were significantly associated with the number of *A. galli *worms in LB. In sheep, where nematode resistance has been a breeding goal much longer than in poultry, *IFNG *variants and markers located in the same chromosomal region as *IFNG *have already been associated with nematode resistance [18-20]. However, any of these polymorphisms were considered to directly influence the investigated trait. The chicken *IFNG *SNP analyzed here is located outside and upstream of the gene promoter [21]. Together with the monomorphic status of this SNP in LSL hens, showing a variance in *A. galli *worm numbers as in LB, it is more likely that its association with the *A. galli *number in LB is due to a linkage with a causal SNP in *IFNG *than influencing the worm number itself. Repeating the association analyses with other hens and with additional neighboring SNPs will be necessary to confirm the genetic influence of *IFNG *on susceptibility to *A. galli *in chickens that is supposed here. However, additional *IFNG *SNPs will be mainly located in non-coding gene regions, since the chicken *IFNG *is known for its high degree of sequence conservation especially in coding regions [21,22].

The existence of only weak linkage to a causal SNP may also be the reason that some SNPs only tended to be associated with one or more of the traits and in only one of the two lines. Therefore, additionally to SNPs in *IFNG*, some of them may be worth studying in further experiments, especially variants of *IL13 *as SNP 25 and other adjacent SNPs.

## Competing interests

The authors declare that they have no competing interests.

## Authors' contributions

GL conceived the study, designed and carried out the molecular genetic and association analyses and drafted the manuscript. MG conceived and designed the infection study, acquired funding and contributed to the interpretation of data. FK acquired, analyzed and interpreted the parasitological data. GE conceived the study, participated in its design and helped draft the manuscript. All authors read and approved the final manuscript.
